# Dyeing Performance and Anti-Superbacterial Activity of Cotton Fabrics Dyed with *Chamaecyparis obtusa*

**DOI:** 10.3390/molecules28186497

**Published:** 2023-09-07

**Authors:** Na-Young Choi, Bog-Im Park

**Affiliations:** 1 Clothing and Textiles Major, Department of Home Economics Education, College of Education, Wonkwang University, Iksan 54538, Jeonbuk, Republic of Korea; 2 Department of Food and Nutrition, School of Food, Kunsan National University, Kunsan 54150, Jeonbuk, Republic of Korea; parkbogim@hanmail.net

**Keywords:** *Chamaecyparis obtusa*, dye, fabric, superbacteria, antibacterial activity

## Abstract

In hospitals, doctors’ and patients’ uniforms, as well as bedding and textiles, can be carriers of superbacteria. This study was conducted to test the anti-superbacterial activity of cotton fabrics dyed with extracts of *Chamaecyparis obtusa* (*C. obtusa*). The dye was extracted by boiling *C. obtusa* in water. The test cotton was mordant-dyed three times with the solution at a 1:17 dyeing bath ratio and at an 8.69% (o.w.f) dye concentration for 15 min at 40 °C. *C. obtusa* dyeing demonstrated a high dyeing affinity in the absence of mordant (K/S value = 14.62). The K/S value of the dyed fabric increased in the order of Cu-mordanted, Fe-mordanted, non-mordanted, and Al-mordanted cotton. Dry cleaning, perspiration and rubbing fastness were determined to be good (Grade 4–5). The dyed fabrics appeared to have a high deodorizing ability compared to the control fabric. They showed not only antibacterial activity against *Staphylococcus aureus* (*S. aureus)* and *Klebsiella pneumoniae* (*K. pneumoniae*), known to be frequently found in fabrics, but also higher antibacterial activity against the superbacteria methicillin-resistant *Staphylococcus aureus* (MRSA) (reduced by 99.7%). These results suggest that fabric dyed with *C. obtusa* extract may be used in clothes and bed linens for inpatients, given its high anti-superbacterial activity. Furthermore, such fabrics may contribute to inhibiting pathogenic infections when used in hospital uniforms or operation gowns for doctors or nurses in hospitals.

## 1. Introduction

Bacteria exist everywhere on Earth, in and around the human body. For example, bacteria are known to adhere to human skin at 10^5^–10^6^ bacteria/cm^2^ or more [1,2,3]. Microorganisms that are found in everyday living spaces include strains largely beneficial to humans, as well as harmful strains that cause infections to the human body, leading to health disorders or potential death [4].

The emergence of superbacteria, in particular, causes mortality and is hard to cure with antibiotics [5]. Since penicillin began to be used in treating bacterial infections, penicillin-resistant bacteria have emerged, and thereafter we have seen antibiotics being developed for the penicillin family, methicillin, and their derivatives [6]. However, deaths by superbacterial infection have recently been increasing, with the emergence of a methicillin-resistant superbacteria, methicillin-resistant *Staphylococcus aureus* (MRSA) [7]. The route of such superbacterial infection includes not only inter-patient infection but also via clothes, bed linens, and various household objects [2]. According to a study recently undertaken by Wiener-Well et al., 65% and 60% of the bacteria found in nurses’ and doctors’ uniforms, respectively, were pathogenic. In addition, 14% and 6% of the bacteria found in nurses’ uniforms and doctors’ uniforms, respectively, were superbacteria [8].

Clothing and textiles can sometimes act as vectors or habitats for pathogenic bacterial infections. In hospitals, doctors’ and nurses’ gowns and uniforms, as well as bedding and textiles, can be carriers of pathogenic bacteria [2,9]. Therefore, in order to prevent infectious diseases and fabric contamination, it is necessary to perform antibacterial processing to suppress the growth of bacteria in fabrics. Antibacterial and deodorizing processing is a fabric processing method that helps to protect the human body and provide a hygienic living environment by suppressing the growth or propagation of bacteria and fungi in fabrics, whilst changing the physicochemical properties of the textile product as little as possible [10]. Recently, general consumers have also begun to think about health- and environment-friendly clothing [11], and there is a growing trend in the textile industry to prefer products with antibacterial and hygienic functions [12].

Natural dyes derived from natural products are recognized for their natural colors, high environmental friendliness and low associated harm to the human body compared to synthetic dyes [13]. Natural dyeing is preferred in order to maintain and improve health. Natural dyes with antibacterial activity against superbacteria seem to have great potential applicability as functional natural dyes in the future. Functional clothing and textiles that inhibit superbacteria will become important in hospitals and the medical field in the future. The aim of our research is to develop antibacterial fabrics that can inhibit superbacteria through natural dyeing. Recently, we reported that natural dyes using *Ulmus davidiana* var. *japonica Nakai* (*U. davidiana*), *Caesalpinia sappan* (*C. sappan*), *Saururus chinensis* (*S. chinensis*), and *Artemisia princeps* (*A. princeps*) can inhibit superbacteria [12].

*Chamaecyparis obtusa* (*C. obtusa*) belongs to the family Cupressaceae. It originates in Japan and was introduced to Korea in the 1900s. It is distributed in the southern regions of the Korean peninsula, such as in Jeonbuk, Jeonnam, Gyeongnam and Jeju [14]. It is expected that the value of using *C. obtusa* for forest healing and health promotion will increase [15]. As the antibacterial [16,17,18] and deodorization [10] properties of *C. obtusa* extracts [19,20,21] have become more widely known, a number of attempts have been made to use it in household items, including in aromatic essential oils, soaps, skin lotions, etc. [22,23]. However, the development of anti-superbacterial cotton fabrics has not yet been attempted.

In this study, we investigated the anti-superbacterial activity of cotton fabric dyed with *C. obtusa,* and also studied its dyeing performance, fastness properties, and deodorization characteristics.

## 2. Results and Discussion

### 2.1. Color Characteristics

**Figure 1:**
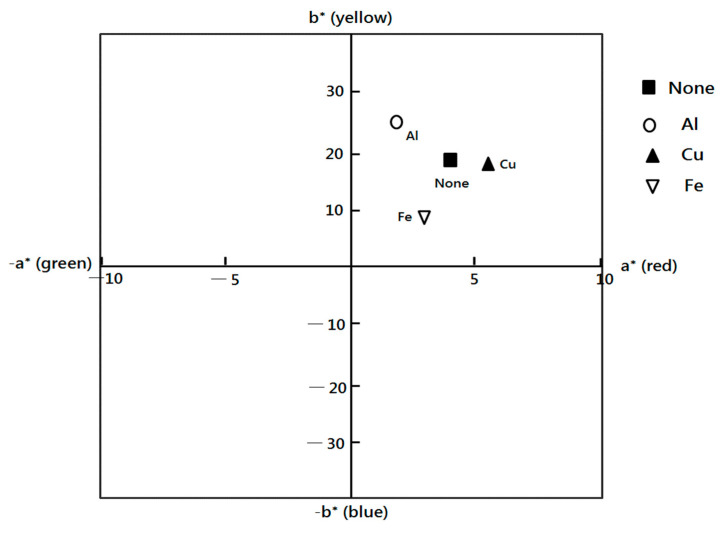
Surface color change, allowing for the color change of the dyed fabric to be examined. +a* direction is a change in color to red, −a* direction indicates a change in color to green, +b* direction means a change in color to yellow, and −b* direction represents a change in color to blue. None, without mordant; Al, aluminum sulfate; Cu, copper sulfate; Fe, ferrous sulfate.

Table 1 and Figure 1 show color changes in the dyed fabric. The color strength (K/S value) of the dyed cotton fabrics, determined according to the mordants, is shown in Table 1. The K/S value of non-mordant-dyed fabric was 14.62, which is considered very high. In general, the K/S value has been reported to be approximately 0.5–2.0 when the cotton fabric is dyed with a natural dye without mordant. A high K/S value implies high color strength, which is equivalent to a high dyeing affinity. Therefore, *C. obtusa* is assessed to have a high dyeing affinity, even without mordant, compared to other natural dyes. The Al-mordanted fabric dyed with *C. obtusa* showed the highest K/S value, 15.38, suggesting the highest color strength. The use of mordant in natural dyeing generally increases the K/S value. In *C. obtusa* dyeing, however, the K/S value has been found to be high in non-mordanted fabric compared to Cu- and Fe-mordanted fabrics. This suggests that *C. obtusa* dyeing without the mordanting process may yield a high color strength, except in cases of Al-mordanted fabric.

L* refers to lightness. The L* decreased after *C. obtusa* dyeing, particularly after the mordanting process. It was the lowest in the Fe-mordanted fabric. In general, the L* value has been reported to decrease in natural dyeing. The result of this experiment showed a reduction in L* similar to that seen in preceding studies [12]. Referring to the red/green coordinates, positive a* values are equivalent to redder shades, while negative values denote green (Figure 1); b* refers to the yellow/blue coordinates, wherein positive values are equivalent to yellow, while negative values denote blue. As regards the changes seen in the colors of fabrics after *C. obtusa* dyeing and mordanting, the a* and the b* values were all positive, suggesting reddish and yellowish colors. It is presumed that the reason for the change in color to reddish and yellowish is the tannin component of the *C. obtusa* leaves being dyed brown by ionic bonds [24]. The red value decreased while the yellow value increased in the A1-mordanted fabric, which showed a high dyeing affinity compared to the non-mordanted fabric. The red value increased while the yellow value decreased in the Cu-mordanted fabric compared to the non-mordanted fabric. Both the red and yellow values decreased in the Fe-mordanted fabric compared to the non-mordanted fabric.

### 2.2. Fastness Properties

The fastness properties of the dyed cotton fabrics are provided in Table 2. The washing fastness was determined as Grade 2–3 only in the Cu-mordanted fabric, which indicates moderate to fairly good, while the rating of Grade 2 seen in the other fabrics indicates moderate. In order to determine the migration of dye from the dyed fabrics to the other fabrics upon washing, non-dyed cotton or silk was washed with the dyed fabrics. The cotton and silk fabrics added were all determined as Grade 4–5, indicating good to very good. It was demonstrated that dye migration did not occur upon washing. Such results show that the use of Cu mordanting on the cotton fabric dyed with *C. obtusa* is the most effective way to retain the color. According to a previous study by Choi et al. (2017), the color fastness of rayon dyed with cypress extract showed similar results [25]. The excellent (not particularly high) washing fastness of the fabric dyed with natural dye is considered one of the unique characteristics of the dye used. Therefore, further studies might be needed to increase the fastness of natural dye in the future.

The dry cleaning fastness was determined as Grade 4–5 in Fe-mordanted fabric, indicating good to very good, but Grade 4 in all other fabrics, which is good (Table 2). The contamination of the test liquid after dry cleaning was determined as Grade 4–5 in all fabrics, indicating good to very good. Such results show that dry cleaning has the benefit of retaining the dye in the cotton fabric dyed with *C. obtusa* compared to washing with water. In addition, when tested with Gromwell extract by Han (2000), the dry cleaning fastness was found to be high compared to natural dye [10].

The light fastness was rated as Grade 3 and Grade 3–4 in most of the *C. obtusa*-dyed fabrics (Table 2). According to the preceding studies, the light fastness is generally rated as Grade 1–3 in natural dye [25,26]. Accordingly, the fabric dyed with natural dye encounters the problem of rapid discoloration in sunlight. However, *C. obtusa* dyeing showed better light fastness than other natural material-dyeing processes.

Perspiration (acid sweat) fastness was determined as Grade 4, which is good, in the non-mordanted fabric (Table 3). However, in terms of discoloration, the mordanted fabric was vulnerable to acid sweat, which decreased its rating to Grade 2–3 or 3. In order to determine the dye migration as it relates to perspiration fastness, a white cotton or white woolen fabric was added to the mix of fabrics tested, and dye migration Grade 4 or 4–5 was achieved, which is good. In relation to alkaline sweat, the non-mordanted fabric and Cu-mordanted fabric were rated as Grade 4, which is good. In terms of dye migration, the addition of a white cotton or white wool fabric to the test fabrics yielded Grade 4 or 4–5, which is very good. The results of dyeing a cotton fabric with cypress were slightly better than those derived in previous studies [25,26] in terms of the degree of discoloration caused by acidic or alkaline sweat.

The wet rubbing fastness was determined as Grade 4 or 4–5, which is very good, in the non-mordanted and AI-mordanted fabrics. These ratings were Grade 4–5, very good, in most of the dyed fabrics (Table 3). These experimental results compared to previous studies [25,26] show that cypress dyeing yields an excellent wet rubbing fastness.

The *C. obtusa* extract-based dye used in this study is highly valued as an excellent eco-friendly and natural dye, with good fastness and with no use of any metal mordant.

### 2.3. Deodorizing Performance

Table 4 shows the deodorizing performance of the non-mordanted and mordanted fabrics. The deodorizing performance over time (from 30 to 120 min) was rated as 70–80% in the raw fabric, 95–98% in the non-mordanted fabric, 96–98% in the AI-mordanted fabric, 96–98% in the Cu-mordanted fabric, and 86–90% in the Fe-mordanted fabric. The deodorizing performance of the non-mordanted fabric was seen to be similar to that of the AI- or Cu-mordanted fabric, but higher than that of the Fe-mordanted fabric. Therefore, dyeing with *C. obtusa* yields a good deodorizing performance regardless of mordanting process used. This seems to be related to the deodorizing function of the active ingredients (such as phytoncide, etc.) in *C. obtusa*.

### 2.4. Antibacterial Activities

**Figure 2:**
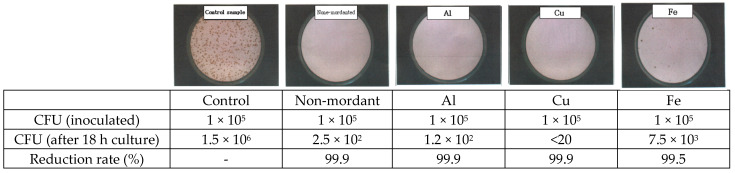
Antibacterial activity of cotton fabrics dyed with *C. obtusa* extracts with different mordants on *S. aureus* (ATCC 6538). Non-mordant, without mordant; Al, aluminum sulfate; Cu, copper sulfate; Fe, ferrous sulfate.

**Figure 3:**
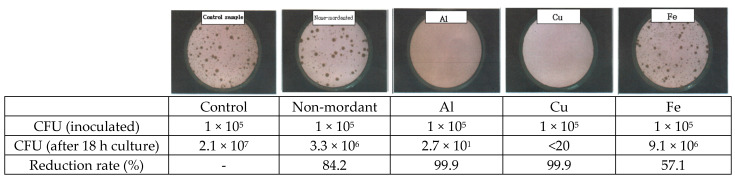
Antibacterial activity of cotton fabrics dyed with *C. obtusa* extracts with different mordants on *K. pneumoniae* (ATCC 4352). Non-mordant, without mordant; Al, aluminum sulfate; Cu, copper sulfate; Fe, ferrous sulfate.

Figure 2 shows the antibacterial activity of cotton fabrics dyed with *C. obtusa* extract against *S. aureus*. At 18 h after the inoculation of bacteria at 1 × 10^5^ CFU into the control cotton fabric, 1.5 × 10^6^ CFU of bacteria was observed. The presence of 2.5 × 10^2^ CFU of bacteria was identified on the non-mordanted cotton fabric, with a 99.9% bacterial reduction rate. The presence of 1.2 × 10^2^ CFU of bacteria was measured on the AI-mordanted fabric, with a 99.9% bacterial reduction rate. The presence of 7.5 × 10^3^ CFU of bacteria was measured on the Fe-mordanted fabric, with a 99.5% bacterial reduction rate. The presence of less than 20 CFU of bacteria was measured on the Cu-mordanted fabric, with a 99.9% bacterial reduction rate, indicating the highest antibacterial activity. Figure 3 shows the antibacterial activity of the cotton fabric dyed with *C. obtusa* extract against *K. pneumoniae*. At 18 h after the inoculation of 1 × 10^5^ CFU of bacteria into the cotton fabric, the presence of 2.1 × 10^7^ CFU of bacteria was observed in the control fabric. The presence of 3.3 × 10^6^ CFU of bacteria was measured on the non-mordanted cotton fabric, with an 84.2% bacterial reduction rate. The presence of 2.7 × 10^1^ CFU of bacteria was measured on the AI-mordanted fabric, with a 99.9% of bacterial reduction rate. The presence of 9.1 × 10^6^ CFU of bacteria was measured on the Fe-mordanted fabric, indicating the lowest bacterial reduction rate of 57.1%. The presence of less than 20 CFU of bacteria was measured on the Cu-mordanted fabric, indicating a 99.9% bacterial reduction rate, which is the highest antibacterial activity.

**Figure 4:**
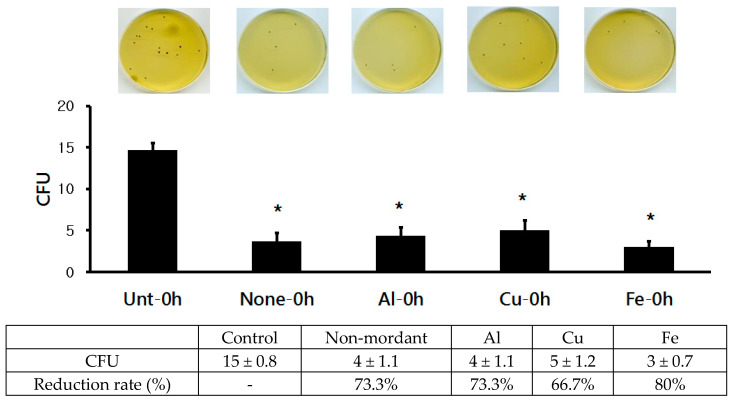
Anti-superbacterial (MRSA ATCC 33591) activity of cotton fabrics dyed with *C. obtusa* extracts with different mordants after 0 h incubation. Control: undyed control cotton fabric; non-mordant, without mordant; Al, aluminum sulfate mordant; Cu, copper sulfate mordant; Fe, ferrous sulfate mordant. Three replicates were made for each concentration of the test extract. * *p* < 0.05 was statistically significant as determined by independent sample t-test for the mean values different from the control group.

In order to measure the anti-superbacterial activity, superbacteria (MRSA ATCC 33591) were inoculated into the dyed fabrics. Bacteria were then immediately collected from the dyed fabrics (at 0 h after inoculation) and inoculated into a solid medium, before being measured for CFU. The results are as follows (Figure 4). On the control fabric, the presence of 15 ± 0.8 cfu/mL of superbacteria was measured. The presence of 4 ± 1.1 cfu/mL of superbacteria was measured on the non-mordanted fabric, indicating a 73.3% bacterial reduction rate. The presence of 4 ± 1.1 cfu/mL of superbacteria was measured on the AI-mordanted fabric, indicating a 73.3% bacterial reduction rate. The presence of 5 ± 1.2 cfu/mL of superbacteria measured on the Cu-mordanted fabric, indicating a 66.7% bacterial reduction rate. The presence of 3 ± 0.7 cfu/mL of superbacteria was measured on the Fe-mordanted fabric, indicating an 80% bacterial reduction rate. Growth of superbacteria was found to be significantly inhibited in the non-, AI-, and Fe-mordanted fabrics (*p* < 0.05) compared to the control fabric. Fe mordanting after dyeing yielded the highest antibacterial activity.

**Figure 5:**
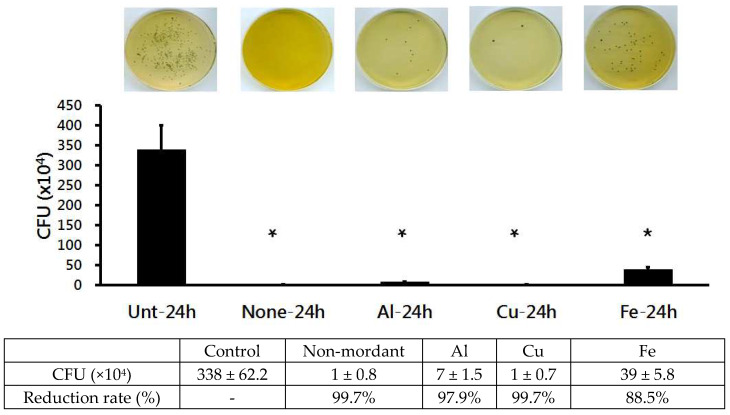
Anti-superbacterial (MRSA ATCC 33591) activity of cotton fabrics dyed with *C. obtusa* extracts with different mordants after 24 h incubation. Control: undyed control cotton fabric; non-mordant, without mordant; Al, aluminum sulfate mordant; Cu, copper sulfate mordant; Fe, ferrous sulfate mordant. Three replicates were made for each concentration of the test extract. * *p* < 0.05 was statistically significant as determined by independent sample t-test for the mean values different from the control group.

After 24 h of culturing of the superbacteria inoculated into the dyed fabrics, bacteria were collected and inoculated into a solid medium, before being measured for CFU. The results are as follows (Figure 5). After the 24 h culturing of bacteria inoculated into the control fabric, the presence of 338 ± 62.2 × 10^4^ cfu/mL of superbacteria was measured. The presence of 1 ± 0.8 × 10^4^ cfu/mL of superbacteria was measured on the non-mordanted fabric, indicating a 99.7% bacterial reduction rate. The presence of 7 ± 1.5 × 10^4^ cfu/mL of superbacteria was measured on the AI-mordanted fabric, indicating a 97.9% bacterial reduction rate. The presence of 1 ± 0.7 × 10^4^ cfu/mL of superbacteria was measured on the Cu-mordanted fabric, indicating a 99.7% bacterial reduction rate. The presence of 39 ± 5.8 × 10^4^ cfu/mL of superbacteria was measured on the Fe-mordanted fabric, indicating an 88.5% bacterial reduction rate. The growth of superbacteria was observed to be significantly inhibited in non-, AI-, Cu-, and Fe-mordanted fabrics (*p* < 0.05) compared to the control fabric. The non-mordanted fabric and Cu-mordanted fabric showed the highest antibacterial effects. These results suggest that dyeing with *C. obtusa* yields high antibacterial activity, even without the mordanting process.

After comparing the bacterial reduction rates in the presence of superbacteria on cotton fabrics dyed with extracts of *U. davidiana*, *C. sappan*, *S. chinensis*, and *A. princeps*, a bacterial reduction rate for C. obtusa of 99.7% was found on the non-mordanted fabrics. This is higher than the bacterial reduction rates achieved by *A. princeps* (18.6%), *U. davidiana* (65.2%), and *S. chinensis* (94.6%), and is almost similar to the bacterial reduction rate of *C. sappan* (99.9%) (12). In AI-mordanted fabrics, the bacterial reduction rate achieved by *C. obtusa* (97.7%) was higher than those achieved by *C. sappan* (54.9%), *U. davidiana* (60.6%), *A. princeps* (70.9%), and *S. chinensis* (94.6%). In Cu-mordanted fabrics, the bacterial reduction rate of C. obtusa (99.7%) was slightly higher than or the same as that of *C. sappan* (99.2%), while it was similar to the rates achieved by *S. chinensis* (99.7%), *U. davidiana* (99.9%) and *A. princeps* (99.9%). In Fe-mordanted fabrics, the bacterial reduction rate of C. obtusa (88.5%) was higher than those of *A. princeps* (24.5%), *C. sappan* (36.9%), *U. davidiana* (42.9%), and *S. chinensis* (77.7%).

The antibacterial activity of *C. obtusa* has previously been reported on [10,16,17,18,22]. However, attempts have not yet been made to develop anti-superbacterial cotton fabrics and clothes via dyeing with *C. obtusa*. To the best of our knowledge, this is the first paper to address the superbacteria-inhibiting effects of *C. obtusa*-dyed cotton fabrics. Superbacteria, one of the major causes of pathogenic infection, are known to jeopardize patient health. In addition, clothes are known to act as potential vehicles of superbacterial infection.

This study suggests that fabrics dyed with *C. obtusa* may be used in clothes and bed linens for inpatients, as they have shown high anti-superbacterial activity. Furthermore, such fabrics may contribute to the inhibition of pathogenic infections when used in hospital uniforms or surgical gowns for doctors or nurses in hospitals.

## 3. Experimental

### 3.1. Materials

C. *obtusa* for use in this experiment was collected from Chungnyeongsan in Jangseong-gun, Jeonnam. Ferrous sulfate (FeSO_4_ 7H_2_O), copper sulfate (CuSO_4_ 5H_2_O), and aluminum sulfate (Al_2_(SO_4_)_3_) (Daejung Chemical Co., Siheung, Republic of Korea) were used as mordants. *Staphylococcus aureus* (*S. aureus)* ATCC 6538 and *Klebsiella pneumoniae* (*K. pneumoniae*) ATCC 4352 were used as the bacteria for the antibacterial tests. These bacteria have been characterized to thrive in fabrics. The methicillin-resistant *Staphylococcus aureus* (MRSA) ATCC 33591 was used for the antibacterial testing on superbacteria [27]. Brain heart infusion (BHI) broth and brain heart infusion (BHI) agar purchased from Becton, Dickinson and Co. (Franklin Lakes, NJ, USA) were used. Oxacillin purchased from Sigma-Aldrich Co. (St. Louis, MO, USA) and Fungizone purchased from Life Technologies Co. (Grand Island, NE, USA) were added to the MRSA culture at 2 μg/mL and 2.5 μg/mL, respectively. A 100% cotton fabric (weave—plain, density—warp 134.8 × weft 132.2, weight—175.4 g/m^2^, count—20.8’s × 21.5’s) was used as the study fabric (Keumsang Textile Co., Jeongup, Republic of Korea).

### 3.2. Extraction of Dye

Sixty kilograms of *C. obtusa* were added to 40 L of water and boiled at 100 °C for 15 h to obtain about 10 L of dye extract solution. The dye extraction process applied to *C. obtusa* is based on the traditional method used in the local *C. obtusa* farm in Jangseong, Jeonnam province, South Korea.

### 3.3. Dyeing

The cotton fabrics used in the experiment were dyed with *C. obtusa* extract at a concentration of 8.69% (o.w.f.), at a 1:17 dyeing bath ratio kept at 40 °C for 15 min. The dyed fabrics were washed 3–4 times in the cold water and naturally dried. This process was repeated twice.

### 3.4. Mordanting

For mordanting, aluminum sulfate mordant at a 5% (o.w.f.) concentration as well as copper sulfate and ferrous sulfate mordants at 3% (o.w.f.) concentration were used. The study fabrics were mordant-dyed with a solution developed in a 1:17 dyeing bath ratio at 40 °C for 15 min. After mordant-dyeing, the study fabrics were washed three times and naturally dried.

### 3.5. Color Measurement

The reflectance values, corresponding CIE coordinates, L*, a* and b* values, and color strength (K/S) values of the dyed fabrics were measured by computer color matching (CCM; Gretag Macbath Color-Eye 7000A, Grand Rapids, MI, USA) using the illuminant D65, with a 10° standard observer [28]. The K/S values were calculated using the Kubelka–Munk equation as follows [29]:

K/S = (1 − R)^2^/2R

(R—the reflectance of the colored fabric, K—absorption coefficient, S—scattering coefficient).

### 3.6. Color Fastness Testing

The washing fastness, dry cleaning fastness, perspiration fastness, rubbing fastness, and light fastness of the dyed fabrics were measured and compared. Detailed methods are provided in the references [30,31,32]. The washing fastness was measured using a Launder-o-meter (Koa Shokai Ltd., Kyoto, Japan) according to KS K ISO 105-C06:2012. Dry cleaning fastness was measured according to KS K 105-D01:2010. Perspiration fastness was measured using a perspiration tester (AATCC, Atlas Electric Device Co, Chicago, IL, USA) according to KS K ISO 105-E04:2010. Rubbing fastness was measured using a Crock meter (AATCC, Atlas Electric Device Co.) according to KS K 0650:2011. Light fastness was measured using a color and color difference meter according to KS K 105-B02:2010.

### 3.7. Deodorization Performance Measurement

Deodorization performance was measured via the gas-detecting tube method. The samples (10 × 10 cm size) were placed in the detector tube and 500 ppm of ammonia was added. The concentration of ammonia in the detector tube was measured over time (at 30, 60, 90 and 120 min) to calculate the deodorization performance.

Deodorization performance (%) = (A − B)/A × 100

(A—gas concentration of blank tube, B—gas concentration of sample tube).

### 3.8. Antibacterial Activity Measurement

The antibacterial activity of the dyed fabric against *S. aureus* ATCC 6538 and *K. pneumoniae* ATCC 4352 was measured according to KS K 0693:2001. The antibacterial activity against superbacteria was measured via a modification of the bioassay method employed by Choi et al. (2015). A cotton fabric sample of 5 × 5 cm^2^ size was prepared and placed in a petri dish (SPL, Pocheon, South Korea) [33]. The cotton fabric was inoculated with MRSA (ATCC 33591) cultured in the BH medium and then incubated in a 37 °C incubator (EYELA, Tokyo, Japan) for 24 h. The bacteria on the cotton fabric were washed with phosphate-buffered saline (PBS) immediately after inoculation at 0 h and after 24 h of culturing, and then spread on the BHI agar plate. After 24 h of culturing the BHI agar plate, the colony-forming unit (CFU) value was measured. The method of CFU counting is provided in detail in [34]. The bacterial reduction rate was calculated via the following:

Bacterial reduction rate (%) = (A − B)/A × 100

(A—bacterial colonies on untreated fabrics, B—bacterial colonies on treated fabrics).

### 3.9. Statistical Analysis

Each set of data has been presented as the mean and standard deviation. Statistical significance was determined using the statistics program SPSS (ver 10.0). The mean values of the study and control groups, respectively, were validated at α = 0.05 using the independent sample *t*-test.

## 4. Conclusions and Perspectives

This study examined the anti-superbacterial, color fastness and deodorization properties of cotton fabrics dyed with *C. obtusa* in relation to different types of mordant, and it compared between the effects of non-mordanting and mordanting.

The K/S values of the fabrics dyed with *C. obtusa* extract were increased in the order of Cu-mordanted (11.37), Fe-mordanted (11.43), non-mordanted (14.62) and Al-mordanted (15.38) fabrics. Dyeing affinity was shown to be the highest in the Al-mordanted fabric. Even without the use of mordant in dyeing, the washing fastness, dry cleaning fastness, perspiration fastness and rubbing fastness were determined to be Grade 4 or 4–5, indicating an excellent rating in general, except for light fastness, which was rated as good. Washing fastness was rated as Grade 4–5 in all fabrics, which is excellent and implies no dye migration to other fabrics. Dry cleaning fastness was rated as Grade 4–5, which is in the range of good to very good, in the Fe-mordanted fabric. Perspiration fastness was rated as Grade 4 or 4–5, which is very good, in most of the dyed fabrics. Rubbing fastness was determined as Grade 4–5—very good—in most of the dyed fabrics. Light fastness was determined as Grade 3–4—moderately good. A high deodorization effect was identified in the fabrics dyed with *C. obtusa* compared to the control fabrics. In the antibacterial test undertaken on the superbacteria, a significant inhibition of bacterial growth was found in both the non-mordanted (99.7%) and mordanted fabrics (88.5–99.7%), with the highest antibacterial effect seen in the Cu-mordanted fabric (99.7%).

These results suggest that cotton fabrics dyed with *C. obtusa* may have antibacterial properties against superbacteria. Further studies are needed to develop medical clothing products for the prevention of superbacterial infection.

## Figures and Tables

**Table 1:** The L*, a* and b* of fabric dyed with *C. obtusa* extracts. The L* is the lightness (0 = black, 100 = white), the a* represents the red/green coordinates (positive values = red, negative values = green), the b^*^ represents yellow/blue coordinates (positive values = yellow, negative values = blue).

Mordants	λ*_max_* (nm)	K/S	L*	a*	b*
None	400	14.62	70.84	4.31	19.65
Al	400	15.38	67.74	2.71	24.25
Cu	400	11.37	56.44	5.51	19.15
Fe	400	11.43	49.34	1.84	8.35

None, dyed without mordant; Al, aluminum sulfate mordant; Cu, copper sulfate mordant; Fe, ferrous sulfate mordant.

**Table 2:** Washing, dry cleaning and light fastness of fabrics dyed with *C. obtusa* extracts with different mordants.

Mordants	Washing			Dry Cleaning		Light
	Color Change	Stain		Color Change	Test Liquid	Color Change
		Silk	Cotton			
None	2	4–5	4–5	4	4–5	3
Al	2	4–5	4–5	4	4–5	3
Cu	2–3	4–5	4–5	4	4–5	3–4
Fe	2	4–5	4–5	4–5	4–5	3–4

None, without mordant; Al, aluminum sulfate; Cu, copper sulfate; Fe, ferrous sulfate. 1 = poor, 2 = moderate, 3 = fairly good, 4 = good, 5 = very good.

**Table 3:** Perspiration and rubbing fastness of fabrics dyed with *C. obtusa* extracts with different mordants.

Mordants	Perspiration						Rubbing	
	Acid			Alkaline			Dry	Wet
	Color Change	Stain Cotton	Stain Wool	Color Change	Stain Cotton	Stain Wool		
None	4	4	4	4	4	4	4–5	4
Al	3	4	4	3	4	4	4–5	4–5
Cu	2–3	4	4	2–3	4	4	4–5	3–4
Fe	3	4	4–5	4	4–5	4–5	4–5	3

None, without mordant; Al, aluminum sulfate; Cu, copper sulfate; Fe, ferrous sulfate. 1 = poor, 2 = moderate, 3 = fairly good, 4 = good, 5 = very good.

**Table 4:** Deodorization activity of cotton fabrics dyed with *C. obtusa* extract.

	Deodorization Activity (%)			
	None	Al	Cu	Fe
30 min	95	96	96	86
60 min	97	97	97	88
90 min	98	98	98	90
120 min	98	98	98	90

None, without mordant; Al, aluminum sulfate; Cu, copper sulfate; Fe, ferrous sulfate.

## Data Availability

The data presented in this work are available in the article.
